# Lack of Class I Vasoreactivity Testing for Diagnosing Patients With Coronary Artery Spasm

**DOI:** 10.1002/clc.70004

**Published:** 2024-08-28

**Authors:** Shozo Sueda, Yutaka Hayashi, Hiroki Ono, Hikaru Okabe, Tomoki Sakaue, Shuntaro Ikeda

**Affiliations:** ^1^ Department of Cardiology Minami Matsuyama Hospital Matsuyma City Ehime Japan; ^2^ Department of Cardiology Ehime Prefectural Niihama Hospital Niihama Ehime Japan; ^3^ Department of Cardiology Ehime University Graduate School of Medicine Touon‐shi Ehime Japan

**Keywords:** acetylcholine, ergometrine, guideline, optimal classification, vasoreactivity testing

## Abstract

**Background:**

Vasoreactivity testing, such as intracoronary acetylcholine (ACh) or ergometrine (EM), is defined as Class I for the diagnosis of patients with vasospastic angina (VSA) according to recommendations from the Coronary Vasomotion Disorders International Study (COVADIS) group and guidelines from the Japanese Circulation Society (JCS).

**Hypothesis:**

Although vasoreactivity testing is a clinically useful tool, it carries some risks and limitations in diagnosing coronary artery spasm.

**Methods:**

Previous reports on vasoreactivity testing for diagnosing the presence of coronary spasm are summarized from the perspective of Class I.

**Results:**

There are several problems such as reproducibility, underestimation, overestimation, and inconclusive/nonspecific results associated with daily spasm. Because provoked spasm caused by intracoronary ACh is not always similar to that caused by intracoronary EM, possibly due to different mediators, supplementary use of these vasoreactivity tests is necessary for cardiologists to diagnose VSA when a provoked spasm is not revealed by each vasoactive agent.

**Conclusions:**

Cardiologists should understand the imperfection of these vasoreactivity tests when diagnosing patients with VSA.

## 1 Introduction

Prinzmetal et al. reported that patients with variant angina experienced ST elevation during attacks in 1959 [1]. Coronary epicardial spasm is the main cause of variant angina. In the early days, as a diagnostic tool, physicians employed intravenous ergometrine (EM) to reproduce coronary artery spasm [2]. However, three cases of death during intravenous EM testing were reported as serious complications in 1980 [3]. The intracoronary injection of EM vasoreactivity test and the intracoronary administration of acetylcholine (ACh) spasm provocation test were reported in 1987 and 1986, respectively [4, 5]. The sensitivity and specificity of intracoronary EM and ACh in patients with variant angina are clinically acceptable. The majority of cardiologists have employed vasoreactivity testing to reproduce daily coronary spasm to diagnose vasospastic angina (VSA) in cardiac catheterization laboratories. In 2008, vasoreactivity testing, such as intracoronary injection of ACh or EM, was defined as Class I according to the Japanese Circulation Society (JCS) guidelines [6]. Furthermore, the Coronary Vasomotion Disorders International Study (COVADIS) group also recommended that spasm provocation tests be defined as Class I when physicians diagnosed the presence of coronary artery spasm in 2017 [7]. Since then, not only Eastern cardiologists but also Western cardiologists have performed these vasoreactivity tests actively to document the presence of coronary epicardial spasm and coronary microvascular spasm in the cardiac catheterization laboratory [8, 9].

In this article, we reconsidered vasoreactivity testing to document daily coronary arterial spasm in the real world.

## 2 Comparison of Classification Grade of Vasoreactivity Testing Worldwide

The JCS and COVADIS groups recommend vasoreactivity testing as Class I for diagnosing VSA. However, the European Cardiology Society (ESC) and American College of Cardiology (ACC)/American Heart Association (AHA) have defined vasoreactivity testing as Class IIb [10, 11]. The reasons for the differences in opinion among the four groups are uncertain. Factors such as the specialized nature of the field, accumulation of experience, or ethnic preferences may contribute to the disparity in the classification of vasoreactivity testing. Eastern cardiologists, such as those in Taiwan, Korea, and Japan, have been interested in the diagnosis of VSA [12, 13], while general cardiologists in Europe and the United States of America, except for those at special institutions, have not been performing vasoreactivity testing in patients with ischemia involving nonobstructive coronary arteries (INOCA) or with myocardial infarction involving the nonobstructive coronary arteries (MINOCA). However, cardiologists should have a worldwide universal classification of vasoreactivity testing to diagnose the presence of coronary spasm, because they are focusing on coronary artery spasm in patients with INOCA and MINOCA.

## 3 Reproducibility of Daily Spasm

According to previous reports, vasoreactivity testing is the gold standard for variant angina to induce coronary artery spasm. Validation studies have verified satisfactory sensitivity and specificity for both the intravenous EM (91% and 97%, respectively) and intracoronary ACh (90% and 99%, respectively) protocols in the diagnosis of spontaneous VSA [2, 14]. Variant angina may represent a very high disease state similar to that of acute coronary syndrome [15]. However, cardiologists may employ these vasoreactivity tests to document the presence of coronary spasm even in patients with low or moderate disease states but not in those with variant angina. Although vasoreactivity testing should contribute to the diagnosis of coronary spasm in patients without variant angina, the sensitivity of vasoreactivity testing in patients with low or moderate disease is not always satisfactory. Standard vasoreactivity testing is not a perfect method, and this procedure may have limitations in reproducing daily coronary spasm in the clinic.

## 4 Underestimation of Daily Spasm

However, even if variant angina occurs with spontaneous ST elevation, spasms may still be provoked by standard vasoreactivity testing in some patients [16]. Furthermore, there may also be negative vasoreactivity test results in some patients with strongly suspected VSA. This misdiagnosis may be related to disease activity, the residual effect of coronary vasodilators, the circadian variation of disease, or imperfect standard vasoreactivity testing. Cardiologists should reconsider the underestimation of provoked spasm by standard vasoreactivity testing.

## 5 Overestimation of Daily Spasm

Patients may also complain of chest pain more often than usual during vasoreactivity testing. The step‐by‐step dose‐up procedure may lead to this phenomenon. The characteristics of each coronary artery spasm may differ. Moreover, the pain or ischemic threshold of coronary vasospasm may differ among patients or among coronary arteries. It may be difficult to reproduce all daily coronary spasms by performing standard vasoreactivity testing. The standard spasm provocation test may have an inevitable problem in the diagnosis of daily coronary spasm in the cardiac catheterization laboratory. Cardiologists should also reconsider the overestimation of standard vasoreactivity testing.

## 6 Inconclusive/Unclassified/Nonspecific Results on Standard Vasoreactivity Testing

European cardiologists may use the terms “inconclusive” or “nonspecific results” for vasoreactivity testing [17, 18]. Positive spasm is defined as usual chest pain, ischemic ECG changes, or > 90% coronary transient constriction. All three issues are necessary for positive provoked spasm, while the presence of two or one issue is defined as inconclusive or unspecific results. According to European studies, inconclusive or nonspecific results were verified in approximately less than 30% of the study patients with INOCA. Furthermore, we also observed unclassified results in 27.4% of patients with angina and nonobstructive coronary artery disease during ACh testing [19]. However, VSA may be mixed in these incomplete cases. According to our previous reports, all three issues (> 90% coronary constriction/usual chest symptom/ischemic ECG change) were recognized in three‐quarters of intracoronary ACh tests and in two‐thirds of intracoronary EM tests when transient coronary constrictions > 90% were observed [20, 21]. This rigorous definition of positive spasm during vasoreactivity testing may expose the fragility of vasoreactivity testing. This may be a limitation of vasoreactivity testing.

## 7 Diversity of Provoked Spasm

The prevalence of provoked spasm in the left circumflex artery (LCX) is less than the prevalence of provoked spasm in the right coronary artery (RCA) and the left anterior descending artery (LAD) after intracoronary injection of ACh and EM [22, 23]. The distribution of serotonergic and muscarinic receptors in the LCX may be less common than that in the RCA and the LAD. Cardiologists should understand the heterogeneity of provoked spasms identified by vasoreactivity testing. Furthermore, the investigation of both coronary arteries may be necessary for cardiologists to strictly document daily coronary spasm.

## 8 Necessity of Investigation of Both Coronary Arteries

Coronary artery spasms are observed in the three coronary arteries, or on the RCA and LCA. Some European physicians perform vasoreactivity testing mainly in the LCA [8]. They perform vasoreactivity testing in the RCA when they cannot find positive results in the LCA by vasoreactivity testing. In contrast, Japanese physicians perform vasoreactivity testing on both coronary arteries if possible [24]. It may be very difficult to administer the bolus intracoronary ACh (20–30 s) without a pacemaker [25]. Cardiologists worldwide reconsider the necessity of investigating both coronary arteries when they diagnose coronary artery spasm. If cardiologists perform vasoreactivity testing in the LCA alone, they may fail to diagnose provoked spasm in approximately 35% (110/314) of patients with INOCA [26]. Cardiologists should perform vasoreactivity testing on both coronary arteries to confidently diagnose coronary spasm.

## 9 Differences Between AChs and EMs

The JCS and COVADIS groups recommend ACh and EM as pharmacological agents to diagnose coronary spasm. However, ACh acts through muscarinic cholinergic receptors, whereas EM acts via serotonergic receptors. Different mediators may have different coronary responses in even the same patients. We previously reported differences between ACh and EM in the induction of provoked coronary spasm [27]. EM provokes more proximal and focal spasm, whereas ACh provokes more distal and diffuse spasm. Approximately half of the vessels (45%) had different coronary responses to the two agents, while only 13% of the provoked spasm vessels had the same site and phenotype. Furthermore, females are more sensitive to ACh, whereas males are more sensitive to EM than females. Vasoreactivity testing may reveal another condition. Cardiologists should reconsider the disparities between pharmacological agents used in cardiac catheterization laboratories.

## 10 Maximum ACh/EM Dose in Standard Vasoreactivity Testing

The COVADIS group and the JCS guidelines recommend that the maximum ACh doses for the RCA and LCA be 50 and 100 μg [6, 7], respectively. In contrast, the JCS guidelines recommend the administration of 40–60 μg of EM in each coronary artery for a few minutes, whereas the COVADIS group does not mention the dose of EM [28, 29]. In the clinic, cardiologists sometimes encounter negative provoked spasm even in patients with variant angina by performing standard vasoreactivity testing. Standard vasoreactivity testing is established for patients with active variant angina who have high disease activity, such as in patients with acute coronary syndrome. To improve the sensitivity of provoked spasm in patients with low or moderate disease conditions, we administered a maximum dose of 80 μg of ACh into the RCA and 200 μg into the LCA [30, 31]. The pseudopositively provoked spasm under the high‐dose injection of ACh and EM may be a residual problem for cardiologists, while there are few people who take care of pseudonegatively provoked spasm by standard vasoreactivity testing. The maximum dose of pharmacological agents needed to reproduce daily coronary spasm may be another limitation.

## 11 Limitations of Single Standard Vasoreactivity Testing as a Diagnostic Tool

Standard vasoreactivity tests, such as intracoronary ACh or EM, may be limited in reproducing daily coronary spasm [32, 33]. Physicians may experience coronary spasm, which is easily provoked by intracoronary ACh but not by EM, whereas cardiologists may encounter coronary spasm, which is easily induced by EM but not by ACh. Furthermore, we may find similar provoked spasms on both intracoronary ACh and EM. The majority of cardiac institutions in the world may employ each vasoactive agent to document coronary spasm in the cardiac catheterization laboratory. At least, supplementary vasoreactivity testing is necessary to verify the presence of coronary spasm [34].

## 12 The Optimal Classification for Vasoreactivity Testing

As mentioned before, vasoreactivity testing has some limitations, such as being Class I for diagnosing patients with VSA. The JCS and COVADIS groups recommend vasoreactivity testing as Class I for diagnosing patients with VSA [6, 7, 28, 29]. While the ESC and ACC/AHA groups define the pharmacological spasm provocation tests as Class IIb [10, 11]. Considering these restrictions, although vasoreactivity testing is defined as Class I, cardiologists should reconsider the restrictions or limitations of vasoreactivity testing for patients with VSA.

## 13 Future Task

Medical therapy and diagnostic tools for patients with VSA are recommended by the ESC, ACC/AHA, COVADIS, or JCS groups. Each VSA guideline has a vasoreactivity testing strategy. Considering ethnic differences and historical transitions, small changes cannot be sufficient. Worldwide guidelines that go beyond the ESC, ACC/AHA, or JCS guidelines are necessary for diagnosing and treating patients with VSA.

## 14 Conclusions

Vasoreactivity testing is the gold standard for variant angina and is defined as Class I according to the JCS and COVADIS groups. However, vasoreactivity testing has some clinical limitations in diagnosing the presence of coronary spasm. Cardiologists should reconsider Class I vasoreactivity testing when diagnosing patients with VSA.

## Conflicts of Interest

The authors declare no conflicts of interest.

## Data Availability

Data sharing is not applicable to this article as no new data were created or analyzed in this study.
